# Isolation and Identification of Lichen Photobionts Collected from Different Environments in North of Portugal and Evaluation of Bioactivities of Their Extracts

**DOI:** 10.3390/foods13111759

**Published:** 2024-06-04

**Authors:** Luís Loureiro, João Morais, Raquel Silva, Joana T. Martins, Pedro Geada, Vítor Vasconcelos, António A. Vicente

**Affiliations:** 1 CEB—Centre of Biological Engineering, University of Minho, 4750 Braga, Portugal; joanamartins@deb.uminho.pt (J.T.M.); pedrogeada@ceb.uminho.pt (P.G.); avicente@deb.uminho.pt (A.A.V.); 2 CIIMAR/CIMAR—Interdisciplinary Centre of Marine and Environmental Research and Department of Biology, Faculty of Sciences, University of Porto, 4169-007 Porto, Portugal; joaopmorais@gmail.com (J.M.); araqssilva@gmail.com (R.S.); vmvascon@fc.up.pt (V.V.)

**Keywords:** lichens, photobionts, *Coelastrella* sp., bioactivity, biotechnology

## Abstract

Lichens are organisms constituted by a symbiotic relationship between a fungus (mycobiont) and a photoautotrophic partner (photobiont). Lichens produce several bioactive compounds; however, the biotechnological exploitation of this organism is hampered by its slow growth. To start studying the possibility of exploiting lichens as alternative sources of bioactive compounds, eighteen lichens were collected in the north of Portugal in order to isolate and study the bioactivity of their photobionts. It was possible to isolate and cultivate only eight photobionts. Three of them, LFR1, LFA2 and LCF3, belong to the *Coelastrella* genus, the other two (LFA1 and LCF1) belong to the *Chlorella* genus and for the remaining three photobionts, LFS1, LCA1 and LCR1, it was impossible to isolate their microalgae. These only grow in consortium with bacteria and/or cyanobacteria. All extracts showed antioxidant activity, mainly at a concentration of 10 mg.mL^−1^. LFS1, a consortium extract, showed the highest antioxidant power, as well as the highest concentration of phenolic compounds (5.16 ± 0.53 mg of gallic acid equivalents (GAE).g^−1^). The extracts under study did not show significant antibacterial activity against *Escherichia coli*, *Listeria* or *Salmonella*. The *Coelastrella* sp. and LFA1 extracts showed the highest hyaluronidase inhibition. The LFR1 extract at a concentration of 5 mg.mL^−1^ showed the highest anti-inflammatory activity (79.77 ± 7.66%). The extracts of *Coelastrella* sp. and LFA1 also showed greater antidiabetic activity, demonstrating the high inhibitory power of α-amylase and α-glucosidase. LFR1 at a concentration of 5 mg.mL^−1^, due to its selective cytotoxicity inhibiting the growth of cancer cells (Caco-2 cells), is a promising anticancer agent.

## 1. Introduction

Lichens are terrestrial organisms formed by a mycobiont, the heterotrophic fungal partner, and a photobiont, the photoautotrophic partner, that have a complex symbiotic relationship [1]. This involves a close physiological and morphological integration between the two organisms, originating the lichen thallus (holobiont) [2]. There is a structural diversity of the lichen thallus, and it adapts in different ways to promote a greater efficiency of light absorption by photobionts. Three morphologies described the three main growth types, the crustose (crust over the substrate, without foliage or shrub), foliose (flattened lobes with leaves) and fructose (like a shrub, with branches) [3]. The structure of the lichen is influenced by evolutionary and ecological factors, namely adaptation to the environment, the mode of transmission of the photobiont and geographic distance [4,5]. The more frequent photobionts belong to the genera *Nostoc*, *Trebouxia* and *Trentepohlia*. The substrates that each genus is most apt to colonize are entirely related to the photosynthetic partner present in the lichen [6].

Lichens are organisms capable of resisting and adapting to severe abiotic stresses, such as UV radiation, oxidative stress, desiccation and extreme temperatures [7,8]. Lichens’ secondary metabolites are identified as key parts in the adaptation to biotic factors and to the presence of other competing plants, herbivorous mammals, predation by insects or exogenous microbes [8]. Over time, the bioactive secondary metabolites of lichens raised great interest due to their biotechnological potential, with the compounds isolated by the dominant fungal partner being mainly studied [9]. However, studies on the cultivation of lichen fungi in bioreactors are still very scarce, and since the growth of lichens is very slow (about 1.5 cm per year), there is still a long way to go regarding the industrial and biotechnological application of lichens’ fungi [10]. Another concern is the quantity of secondary metabolites extracted from lichens, which can range from 0.1% to 10% of the lichen thallus dry weight, thereby presenting a limitation for commercial applications [11]. In contrast, isolated photobionts have a rapid growth rate when isolated from lichens and also have unique adaptations that give them both resistance to high light stresses as well as an increased sensitivity to photo inhibition caused by the lichen thallus drying slowly [12]. However, only in the 1980s did the search for microalgal bioactive compounds extend to other bioactivities beyond antibiotics [13]. The average biomass productivity can reach up to 20 kg.m².year^−1^, with potential for further enhancement through strain selection and process engineering [14].

Reactive oxygen species (ROS) are present in living organisms and induce oxidative damage that can result in chronic diseases such as cancer, diabetes, atherosclerosis and aging [15,16]. Finding sources of antioxidants can help inhibit the oxidative damage caused by ROS [17]. Diabetes is a disease caused by irregularities in glucose metabolism due to a hormonal imbalance of insulin [18]. The inhibition of enzymes responsible for sugar metabolism results in an antidiabetic effect [19]. The best standard treatment of cancer is chemotherapy; however, the medications used have a low level of selectivity for cancer cells and are also cytotoxic to normal cells [20]. In order to react to damage to living tissue, organisms generate a systemic and local response called inflammation [19]. Hyaluronidase is involved in this process, and inhibitors of this enzyme play an important role as an anti-inflammatory and anti-aging agent [21].

Microalgae extracts, despite some tests already performed as substitutes for antimicrobial synthetic compounds on food and feed formulations, still represent an underexplored resource of antimicrobial compounds [22]. Therefore, the evaluation of their role in some of the biological actions of lichens’ extracts (such as antioxidant, antibiotic, antiviral, anti-inflammatory, anti-proliferative and cytotoxic effects) may contribute to their biotechnological and pharmaceutical exploitation. Thus, in the present study, eighteen lichens were collected from different environments in the north of Portugal. The photobiont was isolated and identified. Bioactivities, namely antioxidant, antibacterial, anti-inflammatory, antidiabetic activity and cytotoxicity, were evaluated in the ethanolic extracts of the photobionts.

## 2. Materials and Methods

### 2.1. Materials

Dulbecco’s modified Eagle’s medium (DMEM) with glutamine was obtained from Biowest (Nuaillé, France). Non-essential amino acids (NEAAs) and phosphate-buffered saline (PBS) were purchased from Lonza (Basel, Switzerland). Trypsin/EDTA, penicillin/streptomycin, 3-(4,5-dimethylthiazol-2-yl)-2,5-diphenyltetrazolium bromide (MTT), dimethyl sulfoxide (DMSO), M199 Medium and hygromycin B were purchased from Sigma-Aldrich (St. Louis, MO, USA). Fetal bovine serum (FBS) was obtained from Merck (Darmstadt, Germany). Human colon carcinoma (Caco-2 cell line) and human skin fibroblasts (BJ5ta cell line) were obtained from American Type Culture Collection (LGC Standards, Manchester, UK).

### 2.2. Lichen Sampling

The present study was carried out in Northern Portugal, which has a Csb climate (temperate climate with dry and mild summer), according to the Köppen–Geiger climate classification. Lichens were collected from different areas (coastal, mountainous, city), from different substrates (rock, trees, soils, buildings) and from different altitudes (6 to 180 m). Eighteen lichen sampling units were identified in the field or collected for laboratory identification. A total of 5 g of each lichen sample was collected in sample bags, washed with distilled water, air-dried and stored at room temperature in a desiccator.

### 2.3. Isolation and Purification of Photobiont

Lichens were washed with distilled water at 30 °C for 15 min. After removing all the dirt, the thallus was cut into small pieces and macerated in a mortar in isotonic buffer (0.3 mol.L^−1^ sorbitol in 50 mmol.L^−1^ HEPES pH 7.5) [23]. The homogenate was filtered three times with sterile muslin and centrifuged twice at 2300 rpm for 10 min for debris removal. In order to isolate the photobiont, a gradient centrifugation was performed in a fixed angle rotor (Beckman Coulter Allegra, EVA) [24]. For that, the pellet was resuspended in a 0.25 mol.L^−1^ sucrose solution which was carefully layered onto a KI solution (80% *w*/*v*). The tubes were centrifuged at 4500 rpm for 10 min, and the interphase, where the photobiont was located, was recovered with a Pasteur pipette and subjected to two more centrifugations at 6000 rpm for 10 min [25]. The final pellet containing the photobiont was plated with a sterile loop in culture plates containing solid medium with the following composition (mg.L^−1^): 1100 (NH_2_)_2_CO, 238 KH_2_PO_4_, 204 MgSO_4_.7H_2_O, 40 C_10_H_12_O_8_N_2_NaFe, 116 CaCl_2_.2H_2_O, 0.83 H_3_BO_3_, 0.95 CuSO_4_.5H_2_O, 3.3 MnCl_2_.4H_2_O, 0.17 (NH_4_)6Mo_7_O_24_.4H_2_O, 2.7 ZnSO_4_.7H_2_O, 0.6 CoSO_4_.7H_2_O, 0.014 (NH_4_)VO_3_.

In order to obtain pure microalgae strains, colonies free from other organisms were isolated with a sterile loop from previously cultured plates, and parallel streaks of the colonies were performed in new culture plates. The plates were sealed with parafilm, inverted and incubated at room temperature for 2 weeks. Once isolated colonies had grown, they were transferred to sterile 100 mL Erlenmeyer flasks containing the same culture medium (liquid) and incubated at room temperature for 3 weeks.

### 2.4. Photobiont Identification

#### 2.4.1. Microalgae Culture Conditions

Five microalgae strains previously isolated from different types of lichens and from sampling spots located in the north of Portugal were cultured in 40 mL culture flasks, using Z8 medium [26]. The microorganisms were grown under controlled conditions, at 25 °C with 14/10 h light/dark cycles under a light intensity of 10–30 μmol photons m^−2^ s^−1^. Depending on the strain, after 2–3 weeks of growth, the biomass was harvested for DNA extraction.

#### 2.4.2. Light Microscopy and Morphological Characterization

The morphological features of microalgae strains used in this work were examined and microphotographed using a Leica DMLB light microscope coupled to a Leica ICC50 HD digital camera (Leica Microsystems, Wetzlar, Germany). For each microalgae strain, microphotographs were taken using 400× and 1000× magnifications. Morphometric measurements were then performed using the image analysis software Leica Application Suite version 4.2.0 (Leica Microsystems, Wetzlar, Germany). Cell dimensions were determined measuring at least 20 cells’ diameters for each microalga isolate, along different positions of the slide preparation.

#### 2.4.3. DNA Extraction, Amplification (PCR) and Sequencing

DNA extraction was performed using the NZY Plant/Fungi gDNA Isolation kit (NzyTech, Genes and Enzymes, Lisbon, Portugal) following the manufacturer’s instructions for standard plant DNA extraction. To obtain the 18S rRNA gene sequence, amplification was performed using the external primers 18SF and 18SR [27] (Table 1). PCR reactions were performed in a final volume of 20 μL containing 1× Green GoTaq Flexi Buffer, 2.5 mmol.L^−1^ MgCl_2_, 125.0 mmol.L^−1^ of each deoxynucleotide triphosphate, 1.0 μmol.L^−1^ of each primer, 0.5 U of GoTaq Flexi DNA Polymerase (Promega, Madison, WI, USA), 10 mg mL^−1^ of bovine serum albumin (BSA) and 10–30 ng of template DNA, on a Veriti Dx Thermal Cycler (Invitrogen, Waltham, MA, USA). The PCR conditions for the amplification of 18S rRNA gene fragments were the following: an initial denaturation step for 5 min at 94 °C, followed by 35 cycles, each consisting of a denaturation step of 1 min at 94 °C, an annealing step at 55 °C for 1 min and an extension step for 3 min at 72 °C with the final elongation step at 72 °C for 5 min. The PCR reactions were performed in duplicate. PCR products were separated by 1.5% agarose gel stained with SYBR^®^ safe (Invitrogen, USA), and DNA fragments with the expected size were excised and purified using NZYGelpure (NzyTech, Genes and Enzymes, Lisbon, Portugal) according to the manufacturer’s instructions. Since the sequences were obtained by the direct sequencing of purified amplicons, internal primers 18S402F, 18S895F, 18S919R, 18S1339R [28] (Table 1) were used to improve the quality of sequences. Sequencing was performed at GATC Biotech (Konstanz, Germany), and the nucleotide sequences obtained were manually inspected for quality and assembled using the Geneious 11.1.5 software (Biomatters Ltd., Auckland, New Zealand). The obtained consensus sequences were inserted in the Basic Local Alignment Search Tool (BLASTn) database, and the results were analyzed. Sequences achieved in this study were deposited in the GenBank database under the accession numbers OM985942 to OM985946.

#### 2.4.4. Phylogenetic Analysis

To perform phylogenetic analysis, a total of 81 sequences, 76 of them from microalgae including type and reference strains for *Coelastrella* and *Chlorella* genera retrieved from GenBank (National Center for Biotechnology Information, NCBI, Bethesda, MD, USA) and 5 sequences that were obtained in this work, were used. The selection of sequences chosen to perform this analysis was based on the most recent studies on these genera [29,30,31,32,33]. Multiple sequence alignment was conducted using ClustalW in MEGA7 [34,35], and sequences were manually proofread and edited. Maximum likelihood (ML) analysis was carried out using substitution model GTR + G + I with 1000 bootstrap resampling replicates using the MEGA7 7.0 software [35]. The final phylogenetic tree was edited on iTOL (Interactive Tree of Life) [36].

### 2.5. Ethanolic Extracts Production

The volume of the microalgae culture corresponding to 10 g of dry mass was centrifuged at 6000 rpm for 10 min. The pellet was resuspended in 20 mL of absolute ethanol in a 50 mL sterile Falcon tube. The Falcon tubes were kept for 24 h on a reciprocating shaker at 200 rpm at room temperature. After this period of the dissolution of the bioactive compounds, the tubes were centrifuged at 4 °C, for 15 min at 4000 rpm. The supernatant was collected and filtered with sterile 0.22 µm PES filters. Finally, the extraction solvent was removed on a rotary vacuum evaporator with a water bath at a temperature of 50 °C.

### 2.6. Assessment of Bioactivities

#### 2.6.1. Antioxidant Activity

##### 2,2-Diphenyl-1-Picrylhydrazyl (DPPH) Assay

The DPPH assay was performed according to Ji Sun Youn and collaborators [37], using ascorbic acid as a positive control. A 0.1 mmol.L^−1^ DPPH solution was diluted in ethanol (150 μL) and added to the sample (50 μL). Absorbance was measured at 515 nm after 30 min at room temperature and in the dark. The DPPH radical scavenging activity (*DPPH RSA*) was determined using Equation (1):

*DPPH RSA* (%) = [(*A_control_* − *A_sample_*)/*A_control_*] × 100, (1)

where *A_sample_* is the result of the absorbance of the sample, and *A_control_* is the absorbance of the control (absolute ethanol), upon reaction with ethanol and DPPH solution.

##### Ferric Reducing Antioxidant Power (FRAP) Assay

The FRAP assay performed was described by Benzie et al. [38]; the positive control used was ascorbic acid. The FRAP reagent was prepared using 300 mmol.L^−1^ sodium acetate buffer (pH 3.6), 10 mmol.L^−1^ TPTZ solution (diluted in 40 mmol.L^−1^ hydrochloric acid) and 20 mmol.L^−1^ iron(III) chloride. The FRAP reagent was preheated in a water bath at 37 °C for 20 min, and 3 mL of the reagent was mixed with 100 μL of the sample. Absorbance was measured at 593 nm. The *FRAP Value* was determined from Equation (2):

*FRAP Value* (μmol.L^−1^) = [(*A_sample_* − *A_blanc_*)/(*A_control_* − *A_blank_*)] × 2, (2)

where *A_sample_* is the result of the absorbance of the sample, and *A_control_* is the absorbance of the positive control, and *A_blank_* is the absorbance of the blank, reacted with the FRAP reagent and distilled water.

##### 2,2′-Azino-bis(3-ethylbenzothiazoline-6-sulfonic Acid) (ABTS) Assay

The ABTS method applied was based on the method previous described by Re et al. [39]; ascorbic acid was used as a positive control. The ABTS working solution was prepared from a solution of 7 mmol.L^−1^ ABTS and 140 mmol.L^−1^ potassium persulfate. After 16 h at room temperature and in the dark, the solution (250 μL) was diluted in ethanol (22 mL). A total of 1 mL of this solution was mixed with 50 μL of the sample for 10 min. Optical density was measured at 734 nm. The ABTS radical scavenging activity (*ABTS RSA*) was calculated using Equation (3):

*ABTS RSA* (%) = [(*A_control_* − *A_sample_*)/*A_control_*] × 100, (3)

where *A_sample_* is the result of the absorbance of the sample, and *A_control_* is the absorbance of the control (ethanol solution), reacted with ethanol and ABTS solution.

##### Phenolics Content

Applying the method of Singleton and Rossi [40], the phenolic compounds soluble in the extracts were quantified using the Folin–Ciocalteu reagent and gallic acid as a standard. Thus, 200 μL of samples (1 mg.mL^−1^) or standards for gallic acid was mixed with 1.5 mL of 2% sodium carbonate and 1.5 mL of the Folin–Ciocalteu reagent. After 60 min at room temperature and in the dark, the absorbance (Abs) was measured at 750 nm. The standard curve (*r*^2^ = 0.999) was determined using different concentrations of gallic acid (0, 25, 50, 75, 100, 125, 150 µg.mL^−1^). The total concentration of phenolic compounds in the extracts was obtained using Equation (4):

(4) $$\mathrm{Total}\;\mathrm{phenols}\;(\mathrm{mg}\;\mathrm{GAE}/g\;\mathrm{of}\;{\mathrm{dw}}_{\mathrm{extract}})=\frac{\left(Abs+0.0106\right)}{0.0037}$$

#### 2.6.2. Antibacterial Activity

The microdilution broth susceptibility test was performed according to Saleh and Al-Mariri [41]. In 96-well microtiter plates, three replicates of five dilutions of extracts (0.5, 1.25, 2.5, 3.75 and 5 mg.mL^−1^) and an antibacterial (ampicillin) in LB broth were prepared. In order to conduct this, freshly grown bacteria (*E. coli*; *Listeria*; *Salmonella*) suspensions standardized at 10^6^ CFU.mL^−1^ in LB broth were added to the extracts in a 1:1 proportion. The negative control was carried out without the addition of the pathogen and the positive control with the pathogen but without the addition of extracts. The microplate was incubated on an orbital shaker for 24 h at 37 °C, at 120 rpm. After that period, the absorbance was recorded at 600 nm and the inhibitory levels of the different extracts determined. The lowest concentration that completely inhibited the growth was assessed and interpreted as the minimal inhibitory concentration (MIC) and was expressed in mg mL^−1^.

#### 2.6.3. Anti-Inflammatory and Anti-Aging Potential: Inhibition of Hyaluronidase Activity

The inhibition of hyaluronidase activity was assessed by the method described by Sozmen and coworkers [42]. Phosphate buffer (pH 6.8–7.2) was constituted by 0.1 mol.L^−1^ sodium formate, 0.2 mol.L^−1^ sodium phosphate and bovine serum albumin 0.2 mg.mL^−1^. The reaction mixture was prepared with the phosphate buffer, 20 μL of hyaluronidase enzyme (750 units.mL^−1^), 50 μL of hyaluronic acid (10 mg.mL^−1^) and 20 μL of extract (5 mg.mL^−1^) and was incubated for 30 min at 37 °C. Blanks were performed without enzyme and extracts. At the end of the incubation period, 0.1 mL of alkali borate 0.8 mol.L^−1^ was added and placed in a water bath at 100 °C for 5 min. Lastly, 0.5 mL of 0.2 mol.L^−1^ p-dimethylaminebenzaldehyde was added and the absorbance measured at 580 nm using water as control.

#### 2.6.4. Antidiabetic Activity

##### α–Amylase Inhibition Assay

The methodology applied in α-amylase inhibition assays was described by Ferreira-Santos and coworkers [43], using acarbose as a positive control. Five different concentrations (1, 2.5, 5, 7.5, 10 mg/mL) of the extract were incubated with α-amylase (0.5 mg.mL^−1^) and 1% starch solution at 37 °C for 15 min. Then, 0.1 mL of dinitrosalicylic acid color reagent (96 mmol.L^−1^ 3,5-dinitrosalicylic acid, 5.31 mol.L^−1^ sodium potassium tartrate in 2 mol.L^−1^ NaOH) was added to the reaction and applied for 10 min in a boiling water bath to promote the inactivation of the enzyme. The mixture was diluted 10 times in distilled water, and the maltose in the mixture was quantified at 540 nm. The α-amylase inhibition (%) was calculated using Equation (5):

α-amylase inhibition (%) = [(*A_control_* − *A_sample_*)/*A_control_*] × 100 (5)

##### α–Glucosidase Inhibition Assay

For the α-glucosidase inhibition assay, the applied method was described by Ferreira-Santos and coworkers [43], and acarbose was used as a positive control. Thus, an α-glucosidase solution (10 U.mL^−1^) was incubated with p-nitrophenyl-R-d-glucopyranoside (3 mmol.L^−1^) and with five different (1, 2.5, 5, 7.5, 10 mg/mL) concentrations of the extracts. The mixture was incubated for 15 min at 37 °C, and the reaction was stopped by the addition of a solution of Na_2_CO_3_ (1 mol.L^−1^). In order to assess the p-nitrophenol released and hence the α-glucosidase activity, the optical density was measured at 400 nm. The α-glucosidase inhibitory activity was calculated using the same equation (Equation (5)) used for the α–Amylase inhibition assay.

#### 2.6.5. Cytotoxicity

The extracts’ effect on Caco-2 and BJ5ta cells’ viability was assessed by MTT conversion assay [44,45]. Caco-2 cells were grown in DMEM supplemented with 10% (*v*/*v*) of FBS, 1% (*v*/*v*) of penicillin/streptomycin solution and 1% (*v*/*v*) of NEAAs. BJ5ta cells were grown in 4 parts of DMEM + 1 part of M199 Medium (supplemented with 10% (*v*/*v*) of FBS, 1% (*v*/*v*) of penicillin/streptomycin solution, and 10 µg.mL^−1^ of hygromycin B). Exponentially growing cultures were maintained at 37 °C under a humidified atmosphere of 5% CO_2_. Cells were seeded in 96-well tissue culture plates at 4 × 10^4^ cells.well^−1^, and cells were grown for 24 h to promote cell adhesion. Different extract concentrations diluted in DMEM culture medium (from 0.5 up to 5 mg.mL^−1^) were added to cells. After 24 h of exposure, the medium containing the sample was removed, and the wells were washed with 200 µL of PBS. Then, 100 μL MTT (0.5 mg.mL^−1^) was added to the 96-well plate, and it was incubated during 3 h at 37 °C (5% CO_2_ water-saturated atmosphere). Following the incubation period, the medium was removed, and 200 µL of DMSO was added to each well. Then, plates were placed on an orbital shaker for 30 min to dissolve entirely the formed formazan crystals. The absorbance was read at 570 nm using a Synergy™ HT Multi-mode Microplate Reader (Biotek Instruments, Winooski, VT, USA). Cell viability was expressed as a percentage relative to the control (i.e., untreated cells). A total of four replicate experiments per extract were performed.

## 3. Results and Discussion

### 3.1. Isolation and Identification of Photobionts

The optimized method of photobiont isolation proved to be useful, allowing us to obtain hundreds of photobiont colonies of fructose, foliose and crustose lichens. From small stalks of lichen (10–20 mg), it was possible to isolate microalgae cells.

Of the eighteen lichens collected from different places, substrates, morphologies and altitudes (Table 2), it was possible to isolate the photobiont of eight of them. For some of the lichens, even when applying more abrasive forces and longer times in the maceration process, the number of photobionts obtained was not sufficient for their isolation and purification. Moreover, some of the final microalgal isolates still had fungal or bacterial contamination. This contamination was reduced in the subsequent purification process on culture plates. Of the eight isolated and cultivated photobionts, three of them only grew in consortium with bacteria and/or cyanobacteria, thus hindering their isolation. Although being known that the use of antibiotics would allow for the isolation of the microalgae in these cases, it was considered that the comparison between the bioactivities detected in the cultures of axenic photobionts and consortia cultures could be of added value for this study. Furthermore, it is described that the use of antibiotics can influence the bioactivities presented by microalgae [46], which would be a significant drawback for the purpose of this work.

In the remaining five photobionts, the absence of external contamination and the purity of the microalgal strains were confirmed by the amplification and sequencing of the 18S gene of the rRNA.

**Figure 1:**
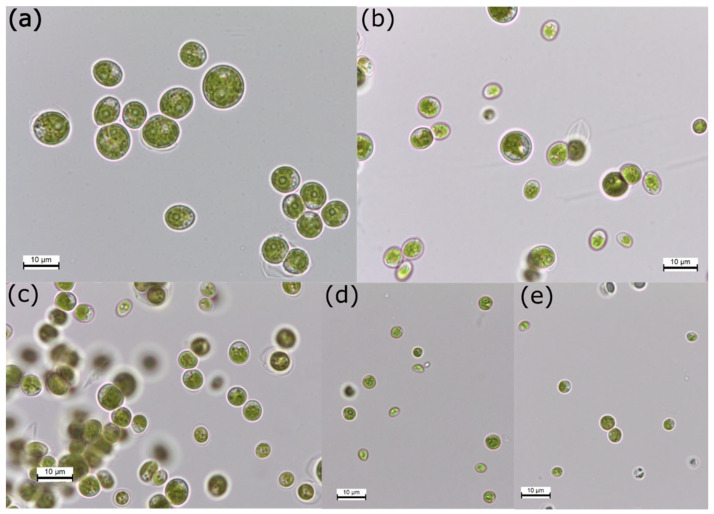
Light microscopy photomicrographs of 5 unialgal strains isolated in this work after two months of growth: (**a**) *Coelastrella* sp. LFR1; (**b**) *Coelastrella* sp. LCF3; (**c**) *Coelastrella* sp. LFA2; (**d**) *Chlorella* sp. LFA1; and (**e**) *Chlorella* sp. LCF1. Scale bar = 10 µm.

The five photobionts isolated were green microalgae with variable forms from spheroidal to ellipsoidal (Figure 1 and Table 3). LFR1, LCF3 and LFA2 belong to the *Coelastrella* genus, and LFA1 and LCF1 were identified as *Chlorella*. The isolated strains of the genus *Coelastrella* (Figure 1a–c) are larger in size than strains of *Chlorella* (Figure 1d,e). Table 3 shows that *Coelastrella* sp. LFR1 has the largest size among the isolated strains (with an average of 8.6 ± 0.7 µm).

**Figure 2:**
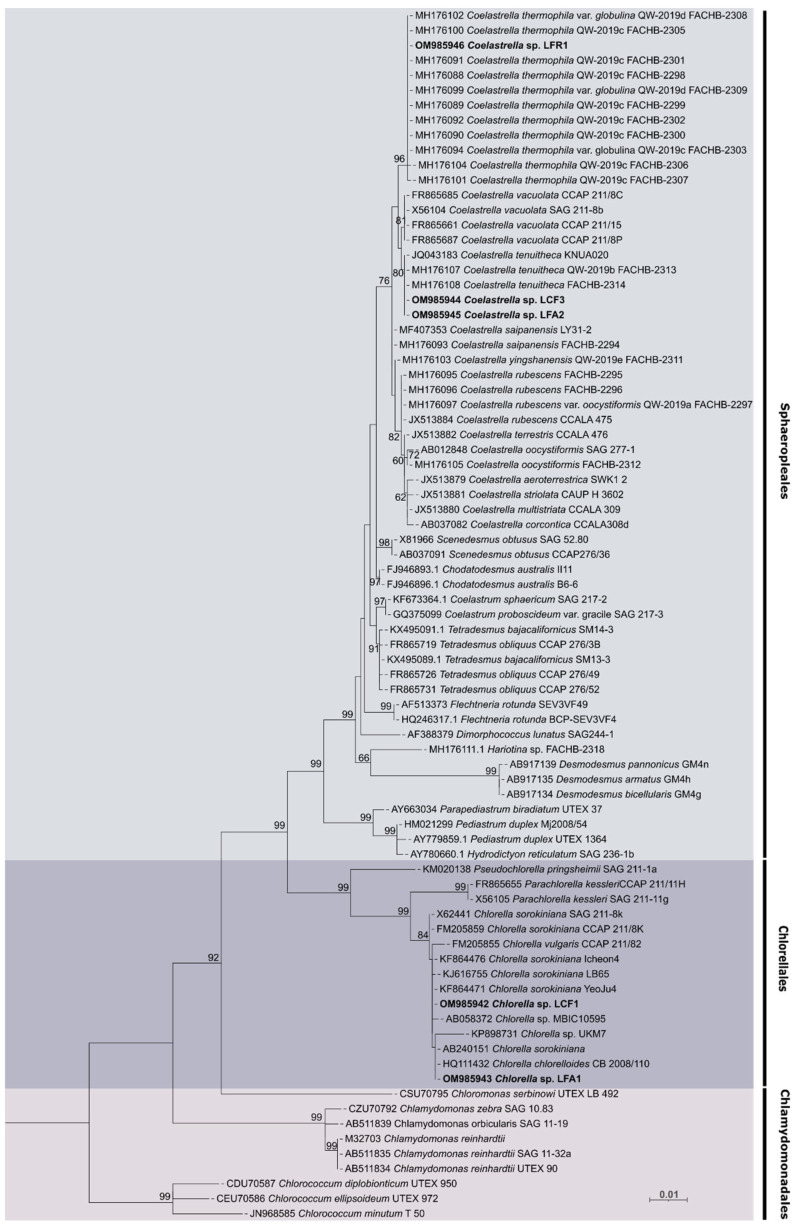
Maximum likelihood (ML) phylogenetic tree based on eighty-one partial 18S rRNA gene sequences of microalgae. Strains isolated in this work are indicated in bold. Different colors represent strain placement in order level. Bootstrap values over 50% are indicated at nodes.

The phylogenetic analysis of the photobionts isolated from lichens was based on the 18S rRNA gene sequence and compared with similar species found on GenBank. Figure 2 shows that the LFR1 isolate has high similarity with other *Coelastrella thermophila* strains. The isolates LCF3 and LFA2 were also grouped in the *Coelastrella* clade but showed greater similarity to the species *Coelastrella tenuitheca*. The three isolates belong to the same order, *Sphaeropleales*. The isolated photobionts LCF1 and LFA1 were identified as belonging to the genus *Chlorella* and consequently to the order *Chlorellalles*.

### 3.2. Bioactivities

#### 3.2.1. Phenolic Content and Antioxidant Activity

The ability to donate a hydrogen atom or an electron to free radicals makes phenolic compounds major antioxidant agents in many plants [47]. These secondary metabolites are also produced by microalgae in response to stressful conditions, thus suggesting the important role these compounds may have in the antioxidative response [48,49]. Thus, to compare and identify which of the photobionts’ extracts isolated from lichens would be a natural source of phenolic compounds, their total phenolic content was determined and expressed as gallic acid equivalents (GAE) per gram of dry weight of extract. The extracts showed an amount of total phenolics that varied between 2.70 ± 0.24 and 5.16 ± 0.53 mg GAE.g^−1^. The eight extracts, namely the extracts of *Coelastrella* sp. (LFR1, LCF3 and LFA2), *Chlorella* sp. (LFA1 and LCF1) and consortia (LFS1, LCA1 and LCR1), did not present amounts of phenolic compounds with significant differences between them. The LFS1 extract showed the highest value of 5.16 ± 0.53 mg GAE.g^−1^. Compared to the phenolic contents of the ethanol extracts analyzed by Goiris and coworkers [50], the photobiont extracts showed higher values than the extracts of *Chaetoceros calcitrans* (1.8 mg GAE.g^−1^) and *Nannochloropsis* sp. (1.4 mg GAE.g^−1^) and values close to the extracts of *Isochrysis* sp. (4.6 mg GAE.g^−1^), *P. tricornutum* (3.8 mg GAE.g^−1^) and *Tetraselmis* sp. (3.8 mg GAE.g^−1^). The comparison between the studies described in the literature and the results obtained must take into account that microalgae growth conditions, such as temperature, nutrient availability and light intensity, strongly influence their phenolic composition [51].

**Figure 3:**
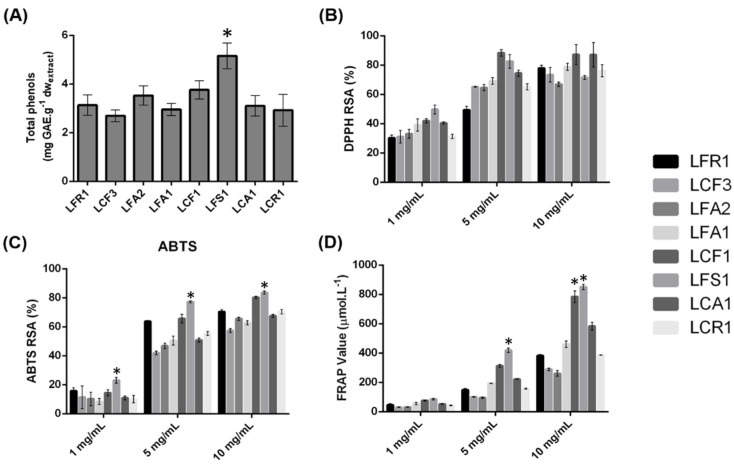
Total phenol content (**A**), antioxidant capacity by DPPH (**B**), ABTS (**C**) and FRAP (**D**) assays of photobiont extracts. * *p* < 0.05 vs. extracts at same concentration.

In order to determine the antioxidant activity of the extracts, the radical scavenging activity was evaluated using the DPPH and ABTS methods and the reducing power using the FRAP method [52,53]. The DPPH and ABTS methods simulate the presence of ROS (reactive oxygen species) through organic radicals that disappear in the presence of antioxidant compounds [48]. FRAP assesses the ability of extracts to donate an electron by reducing the ferric ion (Fe^3+^) to the ferrous ion (Fe^2+^) [54]. The DPPH and ABTS scavenging activity and FRAP value (µmol.L^−1^) were determined for the eight different extracts at concentrations of 1, 5 and 10 mg.mL^−1^ (Figure 3). All extracts showed high DPPH and ABTS scavenging activity at the highest concentration of 10 mg.mL^−1^. The lowest percentage of DPPH scavenging activity for this concentration was obtained for the LFA2 extract of 66.91 ± 1.48% and the highest of 87.21 ± 6.84% for the LCF1 extract. For the extract concentration of 1 mg.mL^−1^, the LFS1 extract showed the highest DPPH scavenging activity of 49.68 ± 3.14%. The same extract was also the one that showed the highest percentage of antioxidant activity for the three concentrations by the ABTS method. For the highest extract concentration of 10 mg.mL^−1^, LFS1 reached the ABTS scavenging activity of 83.61 ± 1.10%. The remaining extracts at the highest concentration (10 mg.mL^−1^), present a percentage between 57.39 ± 1.38 and 80.26 ± 0.90% of ABTS scavenging activity.

The highest reducing power was detected in the 10 mg.mL^−1^ LFS1 extract with a FRAP value of 851 ± 17.90 µmol.L^−1^. All extracts showed a FRAP value greater than 200 µmol.L^−1^ at a concentration of 10 mg.ml^−1^. The extracts of consortia and *Chlorella* sp. were the ones that showed the greatest reducing power. LFS1 seemed to have a greater antioxidant power in most of the methods, which correlates with the composition of phenolic compounds, since it was the extract with the highest concentration of these compounds. It is important to mention that its high antioxidant activity may also be due to the presence of other compounds in the extract, such as carotenoids, polyunsaturated fatty acids and polysaccharides [55]. There is no reference to the antioxidant power of *Coelastrella* sp. in the literature; however, although it was not the extract with the highest bioactivity, it showed high levels of antioxidant power in the different methods, as can be seen in Figure 3.

#### 3.2.2. Antimicrobial Activity

The antibacterial activity of photobionts’ ethanolic extracts was evaluated for extract concentrations of 0.5, 1.25, 2.5, 3.75 and 5 mg.mL^−1^. The effects of higher concentrations have not been evaluated since they present lethal ethanol percentages for bacteria. The minimum inhibitory concentration (MIC) of bacterial growth was estimated against three bacteria (*E. coli*, *Listeria*, *Salmonella*), as shown in Table 4. In this regard, only LCF1 and LFS1 showed some inhibitory effect against *E. coli*; however, their inhibitory concentration will be greater than 5 mg.mL^−1^. The concentration of 5 mg.mL^−1^ of the LCF1 extract showed the greatest inhibitory effect of 33.77 ± 2.53%. *Coelastrella* extracts, LFA1, LCA1 and LCR1, in addition to not having an inhibitory effect, also potentiate bacterial growth. LCF3 showed the greatest stimulating effect on the growth of *E coli*, with a growth 121.62 ± 12.77% higher than the negative control. It was also verified that the higher the extract concentration, the greater the power to stimulate the growth of *E. coli*.

Against *Listeria* and *Salmonella*, the extracts that showed an inhibitory effect were LFR1, LCF1, LFS1 and LCA1, and the minimum inhibitory concentrations for bacterial growth would have to be greater than 5 mg.mL^−1^. LCF1 was the extract that showed the greatest inhibitory effect at the highest concentration tested, 43.22 ± 2.27% and 28.26 ± 0.86% for *Salmonella* and *Listeria*, respectively. As observed in *E. coli*, the remaining extracts did not show inhibition for *Listeria* and *Salmonella*; in contrast, the LCF3, LFA2 and LFA1 extracts promoted bacterial growth greater than 50% at concentrations of 5 mg.mL^−1^. Although bacteria are often considered a contamination in microalgae culture, studies confirm that microalgae and bacteria can synergistically affect each other’s metabolism [56]. These interactions exist in natural habitats, and their interruption often makes it impossible to isolate microalgae in the laboratory [57]. The extracts in this study were obtained from photobionts isolated from lichens, that is, microalgae that live in symbiosis with a fungus and often also with cyanobacteria and other bacteria. Some of the consortia between the photobiont and cyanobacteria and/or bacteria could not be isolated, as is the case of LFS1, LCA1 and LCR1. Thus, the possibility that some of the extracts under study have a potentiating effect on bacterial growth may be due to the existence of organic matter in the extracts, which results in sources of carbon, sulfur, nitrogen or phosphorus for bacteria.

#### 3.2.3. Anti-Inflammatory and Anti-Aging Potential

**Figure 4:**
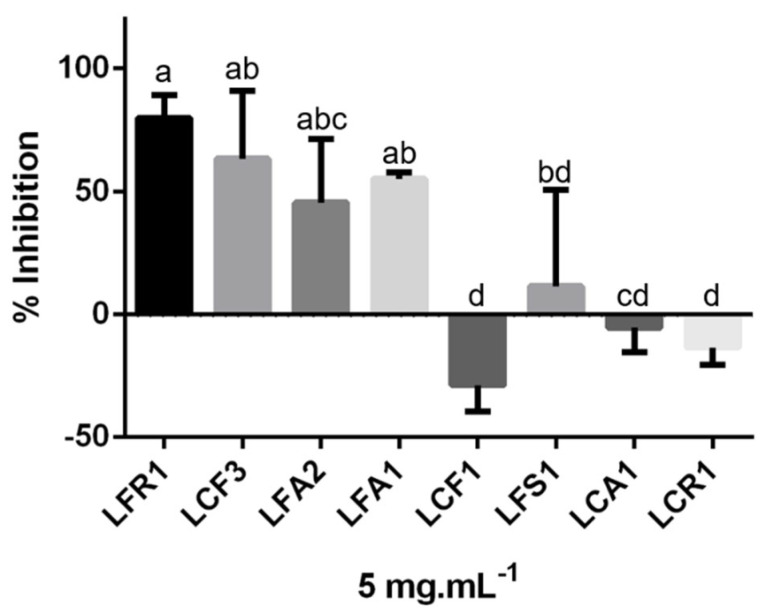
The hyaluronidase inhibitory activity of photobiont extracts. a, b, c, d represent the significant differences among samples in each experiment (*p* < 0.05).

The anti-inflammatory power exhibited by different metabolites present in microalgae and cyanobacteria, such as *Chlorella*, *Dunaliella* and *Phaeodactylum*, has been considered one of the important biological features of those organisms [58]. Hyaluronidase is an enzyme that, in addition to breaking down hyaluronic acid, is also a target enzyme of calcium ions, thereby controlling mast cell degranulation [59]. The evaluation of the hyaluronidase inhibitory activity of the extracts, in addition to evaluating the anti-inflammatory effect, also allows for the anti-aging role of the extracts to be considered. Hyaluronic acid is one of the essential compounds to maintain the elastic properties of the skin, so the inhibition of hyaluronidase will slow down the skin aging process [21]. The inhibitory effects of ethanolic extracts of the eight photobionts at a concentration of 5 mg.ml^−1^ are shown in Figure 4. The extracts of *Coelastrella* sp., LFR1, LCF3 and LFA2, and the extract of *chlorella* sp., LFA1, were the extracts that showed the highest percentage of hyaluronidase inhibition, with no significant differences between them (*p* > 0.05). The LFR1 extract has the highest anti-inflammatory effect of 79.77–7.66%. The LFS1, LCA1 and LCR1 consortium extracts as well as LCF1 did not show considerable hyaluronidase inhibitory effect. Previous studies reveal that low molecules such as oligopeptides, glutamic acid and alanine present in ethanol soluble fractions of the *Chlorella* extracts and polysaccharides included in the insoluble ethanol fractions are responsible for hyaluronidase inhibition [57,58,60]. The present study confirms this inhibitory power of a *Chlorella* sp. extract and even more clearly reports the anti-hyaluronidase activity of *Coelastrella* sp. extracts, which represents a promising anti-inflammatory and anti-aging effect.

#### 3.2.4. Antidiabetic Activity

**Figure 5:**
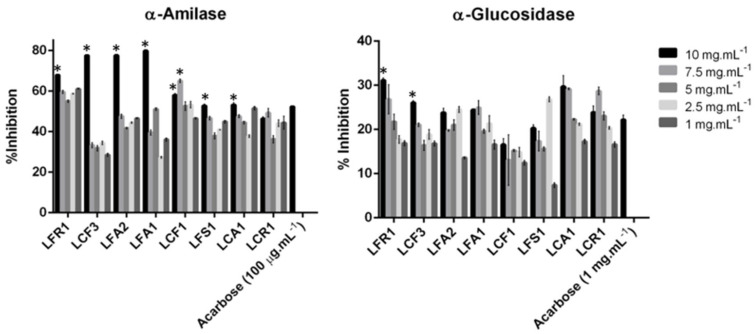
Inhibition of α-amylase and α-glucosidase activity against various concentrations of photobiont extracts. * *p* < 0.05 vs. other concentrations of same extracts.

The evaluation of the activity of specific enzymes responsible for sugar metabolism allows us to infer the antidiabetic power of extracts and/or compounds [61]. The inhibition of α-amylase and α-glucosidase slows the absorption of glucose, as these enzymes are involved in the breakdown of ingested carbohydrates, becoming an effective strategy for diabetes management [62]. Thus, the screening of antidiabetic activity has been performed in five concentrations of the eight extracts of photobionts isolated from lichens by evaluating their inhibition power of α-amylase and α-glucosidase. The results obtained are shown in Figure 5. *Coelastrella* sp. extracts and LFA1 exhibited α-amylase inhibition greater than 67% at a concentration of 10 mg.mL^−1^. The remaining extracts also showed relevant inhibitory activity, and LCR1 was the extract with the lowest percentage of inhibition, 46.27 ± 1.05% for the highest concentration of extract. Previous studies showed that *Chlorella pyrenoidosa* extracts show an α-amylase inhibitory rate of 26.25% up to a concentration of 1 mg.mL^−1^ [63]. All extracts from the present study, at a concentration of 1 mg.mL^−1^, show an inhibitory effect superior to that described by Zheng Sun for this enzyme [63].

The present study also demonstrated that all the extracts’ samples can inhibit α-glucosidase. *Coelastrella* sp. extracts and LFA1, once again, showed high inhibitory power (>23%) at a concentration of 10 mg.mL^−1^. In addition to these extracts, at the same concentration, LCA1 and LCR1 consortium extracts showed an inhibitory effect superior to acarbose (1 mg.mL^−1^), 29.7 ± 2.49 and 23.84 ± 1.50, respectively. The extracts of *Chlorella* sp. and *Porphyridium* sp. were shown to potentially have α-glucosidase inhibition, with a percentage of 12.55 and 12.63% [64]. All lichen photobiont extracts showed inhibitory effects superior to those described above, reinforcing that they possessed moderate α-glycosidase inhibitory activity. From the results obtained for the inhibition of α-amylase and α-glucosidase activity, it is possible to conclude that *Coelastrella* sp., *Chlorella* sp. and consortium extracts can act as effective inhibitors of enzymes relevant for diabetes.

#### 3.2.5. Cytotoxicity

The cytotoxic effect of the photobiont extracts at concentrations of 0.5, 1.25, 2.5, 3.75 and 5 mg.mL^−1^ were evaluated on Caco-2 and Bj5ta cell lines (Table 5). As shown in Table 5, at a concentration of 0.5 mg.mL^−1^, none of the extracts showed toxicity in the Caco-2 cell line, and only LFA1 and LFA2 extracts showed toxicity on fibroblasts (Bj5ta). With the exception of the LCF3 and LFA2 extracts, which keep the percentage of viable cells practically unchanged, the toxicity in Caco-2 also increases progressively with the increase in the concentration of the remaining extracts.

For fibroblasts, noncancerous cells, the extracts showed a behavior similar to that obtained in Caco-2, since with increasing concentration, toxicity also increased. However, in addition to LCF3 and LFA2 that did not show cytotoxicity at a concentration of 5 mg.mL^−1^, in fibroblasts, the LFR1 extract also did not show toxicity. The decrease in the viability of Caco-2, a cancer cell line, and the absence of toxicity on fibroblast cells can indicate the anticancer potential of the LFR1 extract. The anticancer effect may be related to the activation of the apoptotic pathway. Previous studies reported that microalgae and algae extracts are able to activate pro-apoptotic proteins generating cytotoxic effects against tumor cells [65,66,67]. It has been reported that *Chlorella sorokiniana* and *Scenedesmus sp.* extract at a concentration of 500 mg.mL^−1^ and induce 60–70% cytotoxicity in tumor cells by apoptotic mechanisms [68]. Thus, the results of the selective cytotoxic effect of the LFR1 extract are promising in inhibiting the growth of cancer cells without affecting noncancerous cells.

## 4. Conclusions

Of the lichens collected in the north of Portugal, the isolated photobionts LFR1, LFA2 and LCF3 belong to the genus *Coelastrella* sp., LFA1 and LCF1 belong to the genus *Chlorella* sp., and the extracts LFS1, LCA1 and LCR1 only grow in consortium with bacteria and/or cyanobacteria. All extracts showed antioxidant activity at a concentration of 10 mg.mL^−1^. The extracts under study did not show significant antibacterial activity against *E. coli*, *Listeria* and *Salmonella*. *Coelastrella* sp. and LFA1 extracts showed the highest anti-inflammatory and antidiabetic power. LFR1, one of the extracts of *Coelastrella* sp., showed selective cytotoxicity for tumor cells, showing a promising inhibitor of their growth. The determination of the bioactive compounds present in the extracts and which are responsible for the bioactivities studied will be an asset regarding the expansion of research towards the application of these compounds in in vivo, therapeutic and medicinal studies.

## Figures and Tables

**Table 1:** Details of forward (1–3) and reverse (4–6) primers used in this study to amplify the 18S rRNA gene of microalgae strains.

N^o^	Primer Name	Sequence (5′-3′)	Reference
1	18SF	ACCTGGTTGATCCTGCCAG	[27]
2	18S402F	GCTACCACATCCAAGGAAGGCA	[28]
3	18S895F	GTCAGAGGTGAAATTCTTGGAT	[28]
4	18S919R	TAAATCCAAGAATTTCACCTCT	[28]
5	18S1339R	CTCGTTCGTTAACGGAATTAACC	[28]
6	18SR	TGATCCTTCYGCAGGTTCAC	[27]

**Table 2:** Lichens collected for the study, their morphological group, with the respective substrate, sampling site, collection data, latitude and longitude and altitude (m above sea level).

Strain Code	Lichen Morphology	Substrate	Sampling Site	Collection Data	Lat. (N) Long. (W)	Alt. (m)
LFE3	Fruticose	Wood	Esposende, Braga, PT	16 June 2017	41°31′49.3″ 8°46′58.9″	1
LCF2	Crustose	Tree	Barcelos, Braga, PT	16 June 2017	41°29′40.4″ 8°38′45.1″	283
LCE2	Crustose	Wood	Esposende, Braga, PT	16 June 2017	41°31′41.9″ 8°46′53.7″	1
LFE1	Foliose	Tree	Esposende, Braga, PT	16 June 2017	41°32′07.2″ 8°47′06.1″	4
LFF1	Foliose	Rock	Barcelos, Braga, PT	16 June 2017	41°29′40.4″ 8°38′45.1″	288
LFA1	Fruticose	Tree	Braga, PT	18 October 2017	41°33′42.9″ 8°23′46.4″	202
LCA2	Crustose	Rock	Barcelos, Braga, PT	19 October 2017	41°33′19.2″ 8°38′22.1″	121
LFS1	Fruticose	Sand	Esposende, Braga, PT	19 October 2017	41°33′20.0″ 8°47′33.5″	2
LCA1	Foliose	Tree	Barcelos, Braga, PT	19 October 2017	41°33′19.5″ 8°38′23.9″	116
LCF1	Fruticose	Tree	Barcelos, Braga, PT	19 October 2017	41°34′42.8″ 8°33′38.4″	302
LFV1	Fruticose	Tree	Viana do Castelo, PT	15 January 2018	41°48′02.1″ 8°43′57.6″	374
LCF3	Foliose	Tree	Barcelos, Braga, PT	08 February 2018	41°34′41.7″ 8°33′41.5″	291
LFR1	Foliose	Rock	Esposende, Braga, PT	23 April 2018	41°35′27.6″ 8°46′40.9″	201
LFA2	Fruticose	Tree	Esposende, Braga, PT	23 April 2018	41°33′23.8″ 8°45′40.5″	192
LCR1	Crustose	Rock	Esposende, Braga, PT	23 April 2018	41°33′22.2″ 8°45′40.4″	192
LFP1	Foliose	Tree	Póvoa de Varzim, Porto, PT	23 April 2018	41°23′54.7″ 8°45′14.0″	18
LFP2	Crustose	Rock	Póvoa de Varzim, Porto, PT	23 April 2018	41°23′55.0″ 8°45′21.3″	18
LFR2	Fruticose	Rock	Esposende, Braga, PT	01 May 2018	41°33′30.3″ 8°45′34.9″	187

**Table 3:** Cell measures and features from microalgae isolates.

Strain Identification	Cell Dimensions Ø (µm)	Cell Shape	Cell Color
*Coelastrella* sp. LFR1	8.6 ± 0.7 µm	spheroidal to ellipsoidal	Green
*Coelastrella* sp. LCF3	6.4 ± 1.4 µm	spheroidal to ellipsoidal	Green
*Coelastrella* sp. LFA2	6.0 ± 0.9 µm	spheroidal to ellipsoidal	Green
*Chlorella* sp. LFA1	4.5 ± 0.6 µm	spheroidal to ellipsoidal	Green
*Chlorella* sp. LCF1	4.5 ± 0.4 µm	spheroidal to ellipsoidal	Green

**Table 4:** Minimum inhibitory concentration of photobiont extracts against *E. coli*, *Listeria* and *Salmonella*.

	Minimum Inhibitory Concentration (MIC)							
	LFR1	LCF3	LFA2	LFA1	LCF1	LFS1	LCA1	LCR1
Microorganisms	mg.mL^−1^							
*E. coli*	NA *	NA	NA	NA	>5	>5	NA	NA
*Listeria*	>5	NA	NA	NA	>5	>5	>5	NA
*Salmonella*	>5	NA	NA	NA	>5	>5	>5	NA

* No activity.

**Table 5:** Cytotoxic effect of photobiont extracts on Caco-2 (a) and Bj5ta cells (b).

(a)					
**Viability of Caco-2 Cell Line (%)**					
	Photobiont Extracts Concentration				
	0.5 mg.mL^−1^	1.25 mg.mL^−1^	2.5 mg.mL^−1^	3.75 mg.mL^−1^	5 mg.mL^−1^
Control *	100.00				
LFR1	105.96 ± 1.46	112.03 ± 2.06	86.53 ± 6.63	62.40 ± 7.87	24.02 ± 1.82
LFA1	98.44 ± 1.78	91.72 ± 1.01	88.69 ± 2.14	92.22 ± 1.94	13.22 ± 1.49
LCF3	110.95 ± 1.65	76.23 ± 3.41	78.66 ± 6.50	85.99 ±3.97	96.28 ± 8.26
LFA2	104.01 ± 2.61	89.15 ± 7.26	89.98 ± 5.88	93.53 ± 4.71	85.29 ± 5.21
LFS1	117.11 ± 4.37	86.29 ± 1.68	79.42 ± 2.91	60.24 ± 6.21	13.41 ± 1.82
LCA1	98.44 ± 5.77	88.26 ± 4.65	71.77 ± 2.53	51.74 ± 4.65	10.24 ± 0.93
LCF1	117.79 ± 4.29	78.60 ± 4.01	68.21 ± 2.10	34.73 ± 6.83	13.97 ± 2.19
LCR1	117.20 ± 4.94	96.65 ± 7.13	84.48 ± 3.47	67.66 ± 7.56	28.31 ± 6.33
(b)					
**Viability of Bj5ta cell line (%)**					
	Photobiont extracts concentration				
	0.5 mg.mL^−1^	1.25 mg.mL^−1^	2.5 mg.mL^−1^	3.75 mg.mL^−1^	5 mg.mL^−1^
Control *	100.00				
LFR1	98.27 ± 3.15	89.03 ± 5.89	63.52 ± 5.29	103.71 ± 6.08	129.78 ± 3.50
LFA1	82.10 ± 9.62	10.17 ± 1.62	10.96 ± 0.77	27.61 ± 1.45	48.91 ± 0.58
LCF3	102.23 ± 2.50	91.26 ± 2.72	73.88 ± 2.97	63.59 ± 4.15	125.82 ± 3.20
LFA2	80.65 ± 5.77	79.10 ± 2.99	81.23 ± 853	91.21 ± 10.20	101.10 ± 4.88
LFS1	106.53 ± 6.38	109.42 ± 4.52	29.39 ± 2.28	24.59 ± 2.24	39.67 ± 0.63
LCA1	98.44 ± 3.44	77.44 ± 4.93	20.14 ± 1.61	30.63 ± 0.68	40.49 ± 0.48
LCF1	117.12 ± 1.33	106.34 ± 1.39	27.82 ± 0.84	35.30 ± 2.15	67.12 ± 1.93
LCR1	97.32 ± 1.77	84.35 ± 7.00	46.52 ± 3.09	54.81 ± 3.51	62.23 ± 3.32

Values are expressed as mean ± S.E. (Standard Error); * Untreated cells were used as a control.

## Data Availability

The original contributions presented in the study are included in the article, further inquiries can be directed to the corresponding author.
